# Genome-wide identification and expression analysis of serine hydroxymethyltransferase (*SHMT*) gene family in tomato (*Solanum lycopersicum*)

**DOI:** 10.7717/peerj.12943

**Published:** 2022-02-10

**Authors:** Zesheng Liu, Xuejuan Pan, Chunlei Wang, Fahong Yun, Dengjing Huang, Yandong Yao, Rong Gao, Fujin Ye, Xingjuan Liu, Weibiao Liao

**Affiliations:** Gansu Agricultural University, College of Horticulture, Lanzhou, Gansu, China

**Keywords:** Tomato, SHMT, Gene family, Photorespiration, Expression analysis, Abiotic stresses alleviation

## Abstract

Serine hydroxymethyltransferase (SHMT) is one of the most important enzyme families in one-carbon metabolic pathway and photorespiration within plant cells. Recently studies reported the active roles of plant SHMTs in defending abiotic stresses. However, genome-scale analysis of SHMT in tomato is currently unknown. In this study, seven *SHMT* genes were identified in the tomato genome using a genome-wide search approach. In addition, their physicochemical properties, protein secondary structure, subcellular localization, gene structure, conserved motifs, phylogenetic and collinear relationships were analyzed. Our results demonstrated that tomato SHMT members were divided into two group and four subgroups, and they were conserved with the orthologs of other plants. Analysis of *cis*-acting elements showed that each of the *SlSHMT* genes contained different kinds of hormones and stress-related *cis*-acting elements in their promoter regions. Finally, qRT-PCR analysis indicated that *SlSHMT*s were expressed at different levels in different tissues, and they responded to UV, cold, heat, NaCl, H_2_O_2_, ABA and PEG treatments. These results provided definite evidence that *SlSHMT*s might involve in growth, development and stress responses in tomato, which laid a foundation for future functional studies of *SlSHMT*s.

## Introduction

Serine hydroxymethyltransferase (SHMT, EC 2.1.2.1) was first discovered in rat and guinea pig in 1946 by Shemin (1946). With the exploration of protein purification and crystal structure, the property and function of this enzyme have been extensively identified in prokaryotic and eukaryotic organisms (Agrawal et al., 2004). SHMT is an *α*-class pyridoxal-5′-phosphate (PLP)-dependent enzyme which catalyzes the interconversion of serine and glycine. SHMT also can catalyze the generation of methylene tetrahydrofolate (THF), which carries one-carbon groups and takes part in the synthesis of thymidylate, purine and methionine (Zhang et al., 2010).

In mammals, the abnormal expression of *SHMT* may cause tumor growth (Pieroth et al., 2018; Barkla et al., 2014) and neurodegeneration (Troesch, Weber & Mohajeri, 2016; Escande-Beillard et al., 2020) in humans, thus SHMT is regarded as a target protein in genetic control of the corresponding diseases. *SHMT* genes have also been identified in many bacterial systems. More recently, the bacterial SHMTs has been confirmed to involve in alleviating salt and oxidative damage, and this function may be correlated with the photorespiration pathway (Srivastava et al., 2011; Waditee-Sirisattha et al., 2012; Shen et al., 2013; Waditee-Sirisattha et al., 2017; Nogués et al., 2020). Plant SHMT proteins, together with glycine decarboxylase (GDC), manifest an irreplaceable house-keeping function in the primary and secondary metabolisms (Rajinikanth, Harding & Tsai, 2007). In green leaves of plants, mitochondrial SHMT is important for chlorophyll biosynthesis by offering the synthetic precursors glycine and serine. The decreased enzyme activity of mitochondrial SHMT can cause photosynthesis deficiency and growth retardation (Bauwe & Kolukisaoglu, 2003). There also exists a photorespiration pathway in mitochondria of these photosynthetic tissues, which is regulated by phosphoglycolate phosphatase (PGLP), GDC and SHMT (Schwarte & Bauwe, 2007). Except for the release of carbon dioxide (CO_2_), the photorespiratory cycle can also remove the toxic metabolites of photosynthesis produced by ribulose-1,5-bisphosphate carboxylase/oxygenase (RuBisCO) under high oxygen (O_2_) and low CO_2_ environment (Liu et al., 2019a; Liu et al., 2019b). In tomato (*Solanum lycopersicum*), a mitochondrial SHMT protein was found to regulate photorespiration as well as photosynthesis *via* interacting with chaperonin 60 *α*1 (SlCPN60 *α*1), confirming the vital role of SHMTs in adjusting plant metabolisms (Ye et al., 2020). Besides, SHMT activity was also detected in chloroplast, nucleus and cytosol in plants (Zhang et al., 2010; Lakhssassi et al., 2019). The plant-type SHMTs were found to be quite distant from the mammalian and the bacterial SHMTs, suggesting the occurrence of a gene duplication event during the divergence of animals, microorganisms and plants (Agrawal et al., 2004; Sodolescu et al., 2018; Batool et al., 2020).

Plant SHMTs can take part in the defense of pathogen infection. In soybean, GmSHMT08 was found to form a multi-protein complex with the soluble NSF attachment protein GmSNAP18 and the pathogenesis-related protein GmPR08-Bet VI (Lakhssassi et al., 2020). The multi-protein complex then modulated GmSHMT08 activity in maintaining the intracellular redox homeostasis in resistance to soybean cyst nematodes (SCN) (Lakhssassi et al., 2020). In rice, OsSHM1 participated in sheath blight resistance *via* a similar mechanism in soybean (Wang et al., 2021). In addition, a tomato SHMT protein regulated salicylic acid (SA) signaling-dependent basal defense against *Pseudomonas syringae* (Ahammed et al., 2018). Furthermore, adverse environmental conditions can stimulate the accumulation of SHMT in plant cells, such as salt (Mishra et al., 2016; Kito et al., 2017), drought (Liu et al., 2019a; Liu et al., 2019b), chilling (Fang et al., 2020), wound (Hourton-Cabassa et al., 1998) and heavy metal stress (Roth, Roepenack-Lahaye & Clemens, 2006). However, the restrained SHMT activity always comes along with a more severe growth deficiency under abiotic stresses (Moreno, Martín & Castresana, 2005; Liu et al., 2019a; Liu et al., 2019b). SHMT mainly decelerates abiotic stresses through modulating the level of ROS and recovering the impacted cellular metabolism (Ji et al., 2012; Zhou et al., 2012; Fang et al., 2020), which is also assumed to correlate with the photorespiration activity as in cyanobacteria. In *Arabidopsis thaliana*, AtSHMT1 was also found to adjust ABA-induced stomatal closure to reduce salt stress sensitivity (Liu et al., 2019a; Liu et al., 2019b), suggesting a crosstalk mechanism between plant hormones and SHMT proteins in defending abiotic stresses. Nevertheless, there also exists a negative role of SHMT in adapting to the changeable circumstance (Xu & Huang, 2010; Barkla et al., 2014; Fan et al., 2014; Chen et al., 2020a; Chen et al., 2020b), indicating a complex functional allocation of SHMT proteins in different plants. Yet, more explorations are still needed to uncover the accurate mechanism of SHMT proteins in enhancing plant resistance against abiotic stresses.

Tomato (*Solanum lycopersicum*) is a major horticultural crop widely cultivated in the world (Hu et al., 2021). Tomato is favoured by consumers worldwide due to its abundant nutrients (Wang & Liu., 2021). What’s more, tomato is an important model plant in horticultural research, including the study concerning the tolerance of adverse stress (Guo et al., 2021; Li et al., 2021). Hitherto, *SHMT* genes have been identified and characterized in pea, *A. thaliana*, soybean and poplar (Turner et al., 1992; McClung et al., 2000; Wu et al., 2016; Li & Cheng, 2020). As the biochemical properties and the environmental defense mechanism of tomato SHMTs are largely unknown, the genome-wide *SHMT* genes identification and characterization in tomato were investigated in the present study. Here, we analyzed the physicochemical properties, gene structures, evolutionary relationship, collinear relationship, conserved motifs, gene locations, *cis*-acting element distributions and tissue-specific expression patterns of *SHMT* genes in tomato plants. The expression patterns of the tomato *SHMT* genes under different abiotic stress conditions were also detected, and the potential function of SHMT in growth regulation and abiotic stresses alleviation was proposed.

## Materials & Methods

### Genome-wide identification of *SHMT* gene family members in tomato

The tomato genome sequence and annotation information of ITG3.2 were downloaded from the online database Phytozome v12.1 (https://phytozome.jgi.doe.gov/pz/) (Goodstein et al., 2012), and TBtools (Toolbox for ecological battle) v1.0985 software was used to organize and extract SHMT protein sequences by functions of ‘GXF Sequence Extract’ and ‘Batch Translate CDS’ to Protein in TBtools. Sequences of *A. thaliana SHMT* genes were downloaded from the online database SSWISS-PROT/Uniprot (https://www.uniprot.org). The HMM model of the conserved structural domain of SHMT (PF00464) was downloaded from the Pfam database. The preliminary *SHMT* candidate genes were obtained in comparison with the HMM search function of HMMER software. Then, single BLAST of the collected sequences between tomato and *A. thaliana* was performed using the function of ‘BLAST GUI Wrapper’ in TBtools software, and the corresponding bidirectional BLAST was performed using NCBI database (https://www.ncbi.nlm.nih.gov/). ‘Blast Xml to Table’ function in TBtools software was then used to obtain possible gene family members of tomato *SHMTs*. Additionally, the Swiss-Prot/Uniprot database in NCBI was used to further confirm sequences of tomato *SHMT* genes based on the corresponding conserved domains by the ‘Protein BLAST’ function of NCBI.

### Characteristic analysis of tomato SHMT genes

For gene characteristic analysis, molecular weight and isoelectric point (pI) of different *SHMT* genes were conducted by use of the online website Expasy (https://web.expasy.org/compute_pi/). The corresponding gene structure was drawn based on the genome annotation file using the function of ‘Visualize Gene Structure (from GTF/GFF3 File)’ in TBtools software (Chen et al., 2020a; Chen et al., 2020b).

### Phylogenetic analysis of tomato SHMT genes

The SHMT protein sequences of *A. thaliana*, soybean, poplar and tomato were downloaded from the online database phytozome. The multiple sequence alignment of proteins was performed by ClustalW with the Delay Divergent Cutoff value setting as 30 and other options as default. The Mega7.0 software was used to construct a phylogenetic tree of 41 SHMT protein sequences by applying the the Maximum Likelihood method (Kumar, Stecher & Tamura, 2016). In addition, the execution parameters were p-distance and Pairwise deletion, and the number of repeats of Bootstrap repetitions was set as 1000, and the remaining options were set as default. The online website evolview (https://www.evolgenius.info//evolview/#mytrees/clcle/123) was used to further embellish the evolutionary tree.

### Gene structure and chromosomal localization

The gene structure of each member of *SHMT* was analyzed using the ‘Visualize Gene Structure (from GTF/GFF3 File)’ function in TBtools software. The tomato GFF3 file downloaded from the online database Phytozome was prepared to visualize gene locations by Gene Location Visualize from GTF/GFF function in TBtools, Then chromosome location of each tomato *SHMT* gene members was mapped to the tomato genome (Chen et al., 2020a; Chen et al., 2020b). The *SHMT* gene members were renamed according to their chromosome distributions.

### Cis-acting element analysis of tomato SHMT genes

The promoter regions of all the tomato *SHMT* genes were extracted by the function of ‘GXF Sequences Extract’ in TBtools software, and the corresponding promoter sequences of SHMT gene set were further extracted and then submitted to the online PlantCARE (Song et al., 2019) (http://bioinformatics.psb.ugent.be/webtools/plantcare/html/) website to predict different kinds of *cis*-acting elements in *SHMT* gene promoter regions. The *cis*-acting element analysis results of PlantCARE website were collated and simplified, and were conducted to visualization processing by using ‘Simple BioSequence Viewer’ function in TBtools software.

### Conserved motif analysis of tomato SHMT members

The online website MEME (http://meme-suite.org/tools/meme) was used to carry out the conserved motifs information of tomato *SHMT* gene family members (Liu et al., 2019a; Liu et al., 2019b). Then the corresponding motif information and the evolutionary tree information of tomato SHMT family derived from Mega7.0 were combined to be analyzed by the ‘gene structure view (Advances)’ function of TBtools software to visualize the conserved motifs of tomato SHMT members.

### Protein second structure analysis of tomato SHMT genes

The online website prabi (https://npsa-prabi.ibcp.fr/cgi-bin/npsa_automat.pl?page=/NPSA/npsa_sopma.html) was used to analyze the secondary structure of tomato SHMT family proteins and the corresponding information was exported and imaged.

### Collinearity analysis

The online database phytozome was used to determine the genetic relationship between *A. thaliana*, soybean, poplar and tomato. The relative fasta and GFF3 files representing the genetic relationship were downloaded and used for collinearity analysis by ‘Text Merge for MCScanX’ function in TBtools software. Finally, the results were visualized by ‘Multiple Systeny Plot’ function in TBtools software (Chen et al., 2020a; Chen et al., 2020b).

### Plant materials, growth conditions and stress treatments

Tomato (*Lycopersicum esculentum* L. ‘Micro-Tom’) seeds with full grains and consistent size were selected in a 50 mL centrifuge tube, and surface sterilized with 1% NaClO solution for 10 min. The sterilized seeds were put into a 250 mL conical flask filled with 100 mL sterile water, then placed in a HYG-C type shaker and cultured at a rotation speed of 180 r min^−1^ at 25 °C for 3 days. The sterile water was changed once a day. The germinated tomato seeds were planted in a hole dish containing culture soil and placed in a growth chamber for culture. The light intensity in the growth chamber was 250 mol photons m^−2^s^−1^, 26 ± 2 °C for 16 h during the day and 20 ± 2 °C for 8 h at night. The relative humidity was 60%. After 21 days, healthy seedlings of uniform size were selected for subsequent treatments.

As for salt, ABA, H_2_O_2_ and drought stress treatments, the selected seedlings were transferred to 1/2 nutrient solution containing 200 mM NaCl, 100 mM ABA, 10% (w/v) (2.94 M) hydrogen peroxide (H_2_O_2_) and 20% (w/v) PEG6000, respectively. Plants of the control group was grown in the 1/2 nutrient solution without adding other reagents. All the seedlings were grown in the same conditions of a growth chamber. For cold and heat treatments, the seedlings were transferred to the other growth chambers and put into 1/2 nutrient solution under 4 °C and 40 °C without adding other reagents, respectively. Some other selected seedlings were transferred to a growth chamber equipped with 253.7 nm UV-C radiation by a UV-C lamp (TUV PL-S 40 W/4P, Philips, Poland), and the other growth conditions were same as the control. After 0, 6, 12 and 24 h, the aboveground parts of the treated seedlings in each replication were harvested separately, then were frozen with liquid nitrogen and stored at −80 °C, respectively. Each treatment contained three biological replicates, and each replicate consisted of eight seedlings.

Also, the roots, stems and leaves of the 21-day old untreated seedlings were collected for *SHMT* genes expression analysis of the vegetative growth period. The roots, stems, leaves and flowers of the untreated plants were collected at flowering stage (55-day old seedlings), and the corresponding green fruits (25 days after pollination) and mature fruits (45 days after pollination) were also collected (one fruit selected in each plant) to analyze *SHMT* genes expression levels of the reproductive growth period. There were three biological replications with eight plants in each replication collected under the identical experimental condition.

### RNA isolation and qRT-PCR

The methods of RNA isolation and qRT-PCR were done according to Dan et al. (2021) with some modifications. The plant samples in each replication were collected and homogenized in liquid nitrogen to powder. Total RNA was extracted from the homogenized powder by applying a MiniBEST PLANT RNA extraction Kit (TaKaRa). The concentration of the isolated RNA was measured by a NanoDrop 1000 spectrophotometer (Thermo Fisher Scientific, USA). Then 4 µg RNA was used for producing the reverse-transcribed complementary DNA (cDNA) with Dnase I, oligo (dT) primers, dNTPs and M-MLV (TIANGEN) in a 20 µL reaction. A total of 2 µL cDNA from the above reaction was employed as a template to determine the transcript levels of the tested genes with a SuperReal PreMix Plus kit (TIANGEN) on a Roche LightCycler instrument. There were three biological replicates per treatment. The primers used for qRT-PCR were designed using primer premier 5 software, and were listed in File S1. The tomato *ACTIN* gene was used to normalize relative expression levels.

## Results

### Genome-wide identification of *SHMT* genes in tomato

In our study, seven *SHMT* sequences were screened out from the tomato genome database. Our results show that all the seven sequences contain a typical *SHMT* domain (Pfam: PF00464) and belong to the tomato *SHMT* family (File S2). According to the sequential positions of tomato *SHMT* genes identified on tomato chromosomes, the *SHMT* genes were named as *SlSHMT1-SlSHMT7* (Table 1). Then, we analyzed physicochemical properties of the identified *SlSHMT* members. The full length of the open reading frame of the *SlSHMT* genes is between 1,416 bp (*SlSHMT4, SlSHMT7*) and 1,785 bp (*SlSHMT1*), and the corresponding amino acids of tomato SHMTs is between 471 (SlSHMT4, SlSHMT7) and 594 (SlSHMT1). As a result, SlSHMT1 has the longest protein sequence, and its molecular weight is 65521.19 Da, while the molecular weight of SlSHMT4 is lightest (52059.26 Da). Moreover, the isoelectric point (pI) of tomato SHMT members ranges from 6.62 (SlSHMT4) to 8.13 (SlSHMT2). SlSHMT4 is the only acidic (pI < 7) protein in the tomato SHMT family and the rest are alkalescent (pI > 7) (Table 1).

### Protein secondary structure and subcellular localization of *SlSHMT* members

The secondary structure of the proteins encoded by tomato SHMT was analyzed (Table 2). The results show that tomato SHMT family mainly includes alpha helix, random coil and beta turn, with the percentage of 40.07%∼46.91%, 33.01%∼38.72%, 5.89%∼7.02%, respectively.

**Table 1:** Physical and chemical property of SHMT gene family in *Solanum lycopersicum*.

Gene	Gene ID	Gene locus	ORF (bp)	Amino acid	Molecular weight	pI	SHMT domain location
*SlSHMT1*	Solyc01g104000.3.1.ITAG3.2	Chr01	1785	594	65521.19	7.13	137–542
*SlSHMT2*	Solyc02g091560.3.1.ITAG3.2	Chr02	1557	518	57232.43	8.13	56–454
*SlSHMT3*	Solyc04g076790.3.1.ITAG3.2	Chr04	1554	517	57246.39	8.11	55–453
*SlSHMT4*	Solyc05g053810.3.1.ITAG3.2	Chr05	1416	471	52059.26	6.62	12–412
*SlSHMT5*	Solyc08g065490.3.1.ITAG3.2	Chr08	1575	524	56994.42	7.17	78–468
*SlSHMT6*	Solyc12g095930.2.1.ITAG3.2	Chr12	1584	527	57176.71	7.14	81–471
*SlSHMT7*	Solyc12g098490.2.1.ITAG3.2	Chr12	1416	471	52276.55	7.16	12–412

Subcellular localization of tomato SHMTs showed that SHMT genes are mainly distributed in chloroplasts, mitochondria, cytoskeletons and cytoplasms (Table 3). However, the distribution of each member in different cellular parts is different. SlSHMT1, SlSHMT3, SlSHMT5 and SlSHMT6 in tomato *SHMT* family were found to be chloroplast-localized. Only SlSHMT2 and SlSHMT3 are distributed in mitochondria. SlSHMT4 and SlSHMT7 were found to mainly localize in cytoskeletons, and the cytoplasms-localized proteins are SlSHMT1, SlSHMT2 and SlSHMT4. The localizations of tomato SHMTs in nucleus and cytoplasms are relatively low.

**Table 2:** The secondary structure of *SHMT* gene family in *Solanum lycopersicum*. Blue indicates alpha helix; Green indicates beta turn; Red indicates extended strand; Pink indicates random coil.

Protein	Alpha helix (%)	Beta turn (%)	Random coil (%)	Distribution of secondary structure elements
SlSHMT1	40.07	5.89	38.72	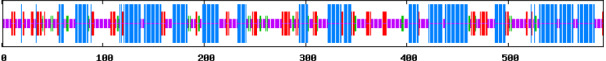
SlSHMT2	46.91	6.95	33.01	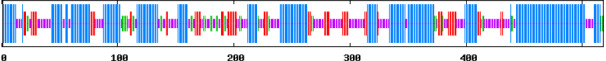
SlSHMT3	45.26	6.58	35.01	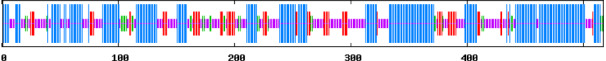
SlSHMT4	44.80	6.16	35.24	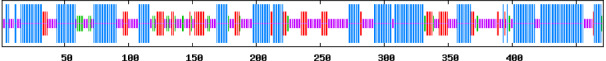
SlSHMT5	42.75	6.49	36.26	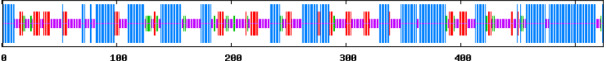
SlSHMT6	42.13	7.02	36.05	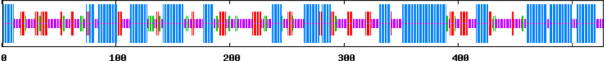
SlSHMT7	44.59	5.94	36.94	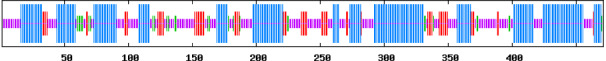

### Gene structure analysis of *SlSHMT* genes

**Figure 1:**
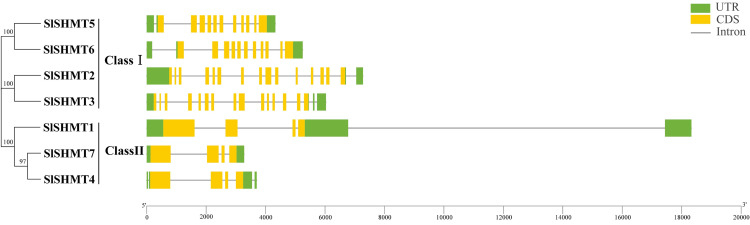
Exon-intron structure of *SHMT* gene family in tomato. The evolutionary tree was constructed based on the full length of tomato SHMT protein sequences using MEGA7.0. The exon-intron graph of tomato SHMT genes was drawn using TBtools software.

According to the information of phylogenetic analysis, tomato SHMT proteins are divided into two groups (Fig. 1). In detail, SlSHMT2, SlSHMT3, SlSHMT5 and SlSHMT6 are classified into Class I, and SlSHMT1, SlSHMT4 and SlSHMT7 are classified into Class II. Gene structure analysis is regarded as a very effective method to determine gene function and reflect the phylogenetic relationship between genes. Therefore, the gene structure of tomato *SHMT* family members were evaluated by using a TBtools software (Fig. 1). Our results show that *SlSHMT5* and *SlSHMT6* (Class I) both contain 11 exons and 11 introns. *SlSHMT2* and *SlSHMT3* (Class I) possess 15 exons and 15-16 introns. Thus, *SlSHMT2* and *SlSHMT3* were more similar in their gene structures than *SlSHMT5* and *SlSHMT6* in Class I. In Class II, all the members contain 4 exons, and *SlSHMT1*, *SlSHMT4* and *SlSHMT7* have 4, 4, and 3 introns, respectively. Gene structures of *SlSHMT4* and *SlSHMT7* were quite similar with each other, and *SlSHMT1* gene possessed relative longer exons, introns and UTR regions. In all, the distributions of exon-intron in the same protein group are similar, and the sequence lengths of *SHMT* genes vary between different subfamilies. This difference is mainly caused by the sequence length differences of non-coding regions and the amounts of introns. Thus, the two subfamilies may have undergone functional differentiation during evolution.

**Table 3:** Subcellular localization prediction of *SHMT* gene family in *Solanum lycopersicum*.

Gene	Gene ID	Chloroplast	Nucleus	Plasma membrane	Mitochondria	Cytoskeleton	Cytoplasm
*SlSHMT1*	Solyc01g104000.3.1.ITAG3.2	7	2	–	–	1	4
*SlSHMT2*	Solyc02g091560.3.1.ITAG3.2	1	–	–	6	–	7
*SlSHMT3*	Solyc04g076790.3.1.ITAG3.2	8	–	–	6	–	–
*SlSHMT4*	Solyc05g053810.3.1.ITAG3.2	2	2	–	–	6	4
*SlSHMT5*	Solyc08g065490.3.1.ITAG3.2	11	1	1	–	1	–
*SlSHMT6*	Solyc12g095930.2.1.ITAG3.2	13	–	–	–	–	1
*SlSHMT7*	Solyc12g098490.2.1.ITAG3.2	–	3	–	–	8	3

### Phylogeny and Collinearity analysis of *SlSHMT* members

**Figure 2:**
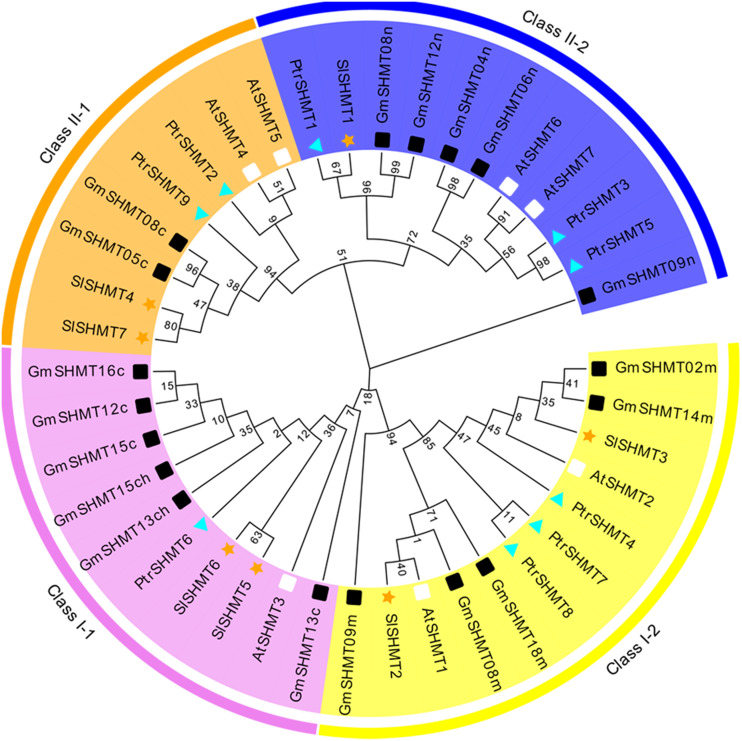
The unrooted phylogenetic tree of *SHMT* gene family in *Solanum lycopersicum*, *Glycine max*, *Populus trichocarpa* and *A. thaliana*. The maximum likelihood method was used to construct phylogenetic tree containing seven tomato, seven *A. thaliana* (At), nine poplar (Ptr), and 18 soybean (Gm) SHMT proteins. The four subgroups are colored differently. The four differently-colored shapes represent SHMT proteins from four species. The orange pentacle, white rectangle, blue triangle, black rectangle represent tomato, *A. thaliana*, poplar, and soybean SHMT proteins, respectively. The number on the node in the phylogenetic tree represents the percentage of trustworthiness of the branch in the bootstrap validation.

In order to further understand the functions and characteristics of tomato *SHMT* family genes, we compared the full-length sequences of 41 SHMT proteins in *A. thaliana*, soybean (*Glycine max*), poplar (*Populus trichocarpa*) and tomato (Fig. 2 and File S3). The 41 SHMT proteins of these plants are divided into two groups: Class I and Class II, which is parallel with the classification in Fig. 1. The two groups are further divided into four subgroups: Class I-1, Class I-2, Class II-1 and Class II-2. There are two members of tomato SHMT family (SlSHMT5 and SlSHMT6) in Class I-1, two members (SlSHMT2 and SlSHMT3) in Class I-2, two members (SlSHMT4 and SlSHMT7) in Class II-1, and one member (SlSHMT1) in Class II-2. In addition, the homology coefficient of SlSHMT6 and SlSHMT7 is very high, and they are located on the same chromosome, indicating that gene replication may take place between them.

**Figure 3:**
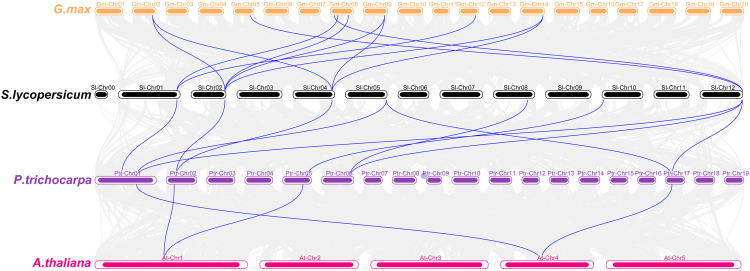
Collinearity analysis of *SHMT* gene family in *S. lycopersicum*, *G. max*, *P. trichocarpa* and *A. thaliana*. The blue lines delineate the syntenic *SHMT* gene pairs.

Then, we also explored the collinear relationship between tomato *SHMT* genes and the related genes of the three typical plants *A. thaliana*, soybean and poplar based on the evolutionary relationship among different species (Fig. 3). The results of collinearity analysis showed that seven tomato genes are collinear with nine soybean genes, six poplar genes and two *A. thaliana* genes. There are 11 homologous pairs between tomato and soybean, 10 homologous pairs between tomato and poplar, and four homologous pairs between poplar and *A. thaliana*. The above results indicated that tomato SHMTs are closely related to other SHMT members in the above three plant species.

### Conserved motifs analysis of tomato SHMT proteins

**Figure 4:**
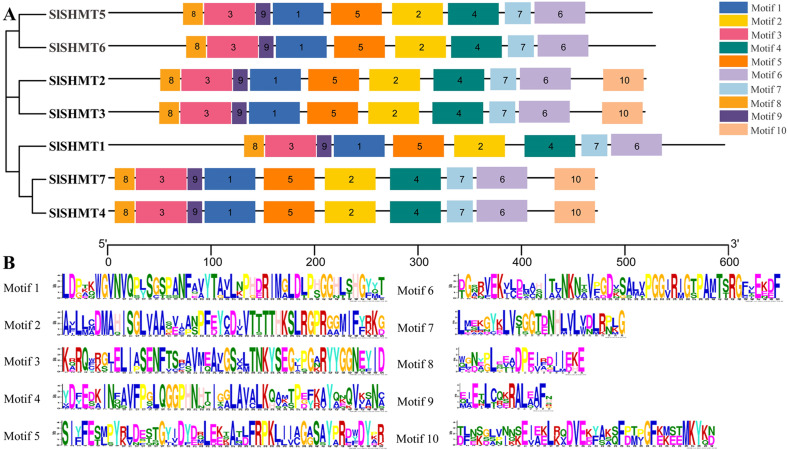
The motif composition and distribution of tomato SHMT proteins. (A) Colored boxes represent different conserved motifs, and motifs 1-10 are shown in (B). (B) Amino acid sequences of different conserved motifs displayed by stacks of letters at each position. The total height of the stack represents the information content of the relative amino acid in the position of each letter in the motif in bits. The height of the individual letter in a stack is calculated by the probability of the letter at that position times the total information content of the stack. X- and *Y*-axis represents the width and the bits of each letter, respectively.

The online site MEME was used to study the conserved region of tomato SHMT proteins. In the recent studies, 10 conserved motifs were found in tomato SHMT proteins (Fig. 4). The sequence information of the identified conserved motifs was listed in Table 4, and the length of each motif ranges from 15 to 50 amino acids. Using TBtools software, we found that the tomato SHMT proteins belonging to the same subfamily in the evolutionary tree contain the similar or identical composition of motifs. For example, all the members of Class I-1 (SlSHMT5 and SlSHMT6) lack the tenth conserved motif, while the members of Class I-2 (SlSHMT2 and SlSHMT3) contain this motif. The members of Class II-2 (SlSHMT1) contain nine motifs as that of class I-2, and the motif locations are significant different with that in Class II-1. SlSHMT4 and SlSHMT7 in Class II-1 contain all the 10 conserved motifs. Furthermore, the sequence of motifs in each subfamily is almost the same. These results are in agreement with the phylogenetic analysis of the tomato SHMT proteins, which further suggest that these 4 subgroups may have developed functional difference during evolution.

**Table 4:** Details of the 10 conserved motifs of *Solanum lycopersicum* SHMT proteins.

Motif	Width (aa)	Motif Sequence
*Motif 1*	50	LDPKKWGVNVQPLSGSPANFAVYTAVLNPHDRIMGLD LPHGGHLSHGYYT
*Motif 2*	50	ALLLCDMAHISGLVAASVIANPFEYCDIVTTTTHKS LRGPRGGMIFYRKG
*Motif 3*	50	KQRQFRGJELIASENFTSRAVMEAVGSALTNKYSEG LPGARYYGGNEYID
*Motif 4*	50	YDFEDKINFAVFPGLQGGPHNHTIGGLAVALKQAKT PEFKAYQEQVKANA
*Motif 5*	50	SIYFESMPYRLDESTGYIDYDRLEKSATLFRPKLIIA GASAYPRDWDYPR
*Motif 6*	50	DGSRVEKVLDLAHITLNKNSVPGDKSALVPGGIRIGT PAMTSRGFVEKDF
*Motif 7*	26	LMEKGYKLVSGGTDNHLVLVDLRPLG
*Motif 8*	20	WGNEPLEEADPEIADIIEKE
*Motif 9*	15	ZIETLCQKRALEAFH
*Motif 10*	40	DFNKGLVNNKEIEELKQDVEKYAKQFPTPGFEKEEMKYKD

### Chromosomal location of tomato *SHMT* genes

**Figure 5:**
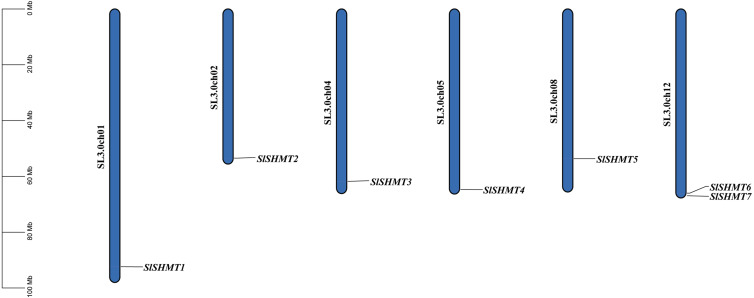
The distribution of *SHMT* gene family members of chromosomes in *Solanum lycopersicum*.

Using tomato genome annotation information and TBtools software, we visualized the chromosomal distributions of tomato *SHMT* gene family members (Fig. 5). As could be seen from Figs. 5, seven genes of tomato *SHMT* family are unevenly distributed on six chromosomes (Chr.1, Chr.2, Chr.4, Chr.5, Chr.8 and Chr.12), and the number of genes on each chromosome is unrelated to the chromosome size. Among the tomato *SHMTs*, *SlSHMT1* is distributed on chromosome 1, *SlSHMT2* is distributed on chromosome 2, *SlSHMT3* is distributed on chromosome 4, *SlSHMT4* is distributed on chromosome 5, *SlSHMT5* is distributed on chromosome 8, *SlSHMT6* and *SlSHMT7* are both distributed on chromosome 12. There are no *SHMT* members on the chromosome 3, 6, 7, 9, 10 and 11. Most *SlSHMT* genes are located on the proximal or distal end of the tomato chromosomes.

### *Cis*-acting element analysis of tomato *SHMT* genes

**Figure 6:**
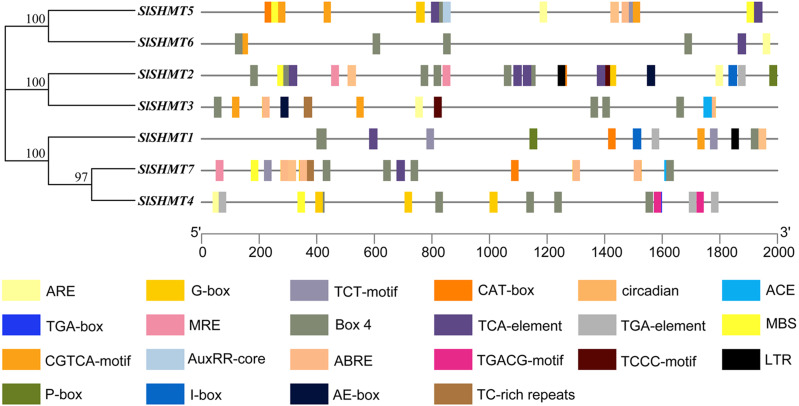
The distribution of *cis*-acting elements in *Solanum lycopersicum SHMT* genes.

**Figure 7:**
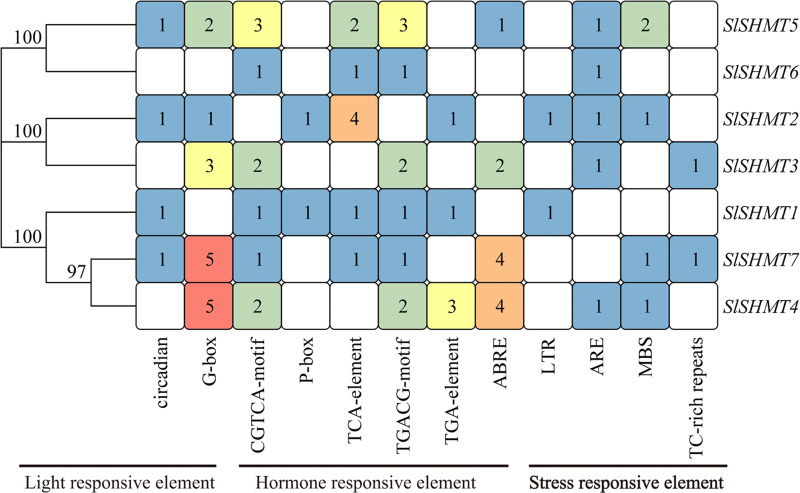
The number of *cis*-acting elements in *Solanum lycopersicum SHMT* genes.

In the current study, 46 types of elements were analyzed in the promoter region of *SlSHMT* genes. The predicted *cis*-acting elements were classified as stress responsive elements, light responsive elements, and hormone responsive elements according to the function and action of these elements (Table 5). The results show that *SlSHMT* members contain 7-23 *cis*-acting elements in their promoter region (Fig. 6 and File S4). The stress responsive elements include LTR, ARE, MBS and TC-rich repeats. The light responsive elements include Circadian and G-box. Some hormone responsive elements are also found in most of the *SlSHMT* genes, including CGTCA-Motif, P-box, TCA-Element, TGACG-Motif, TGA-Element, and ABRE (Fig. 7).

**Table 5:** Summary of cis-acting elements of *Solanum lycopersicum SHMT* genes.

Element	Sequence	Description
Circadian	CAAAGATATC	*cis*-acting regulatory element involved in circadian control
G-box	CACGAC	*cis*-acting regulatory element involved in light responsiveness
CGTCA-motif	CGTCA	*cis*-acting regulatory element involved in the MeJA-responsiveness
P-box	CCTTTTG	gibberellin-responsive element
TCA-element	CCATCTTTTT	*cis*-acting element involved in salicylic acid responsiveness
TGACG-motif	TGACG	*cis*-acting regulatory element involved in the MeJA-responsiveness
TGA-element	AACGAC	auxin-responsive element
ABRE	ACGTG	*cis*-acting element involved in the abs*cis* ic acid responsiveness
LTR	CCGAAA	*cis*-acting element involved in low-temperature responsiveness
ARE	AAACCA	*cis*-acting regulatory element essential for the anaerobic induction
MBS	CAACTG	MYB binding site involved in drought-inducibility
TC-rich repeats	ATTCTCTAAC	*cis*-acting element involved in defense and stress responsiveness

As can be seen from Fig. 7, G-box elements are mostly distributed in *SlSHMT3*, *SlSHMT4*, *SlSHMT5* and *SlSHMT7*. CGTCA-Motif elements are mostly distributed in *SlSHMT3*, *SlSHMT4* and *SlSHMT5*. TCA-element elements are mostly distributed in *SlSHMT2* and *SlSHMT5*. TGACG-motif elements are mostly distributed in *SlSHMT4*. ABRE elements are more distributed in *SlSHMT3*, *SlSHMT4* and *SlSHMT7*. Finally, MBS elements are mostly distributed in *SlSHMT5*. Our analysis of *cis*-acting elements shows that most of the *SlSHMT* genes may play an important role in stress, light and hormone responses.

### Expression analysis of tomato *SHMT* genes in different organs of different growth stages

**Figure 8:**
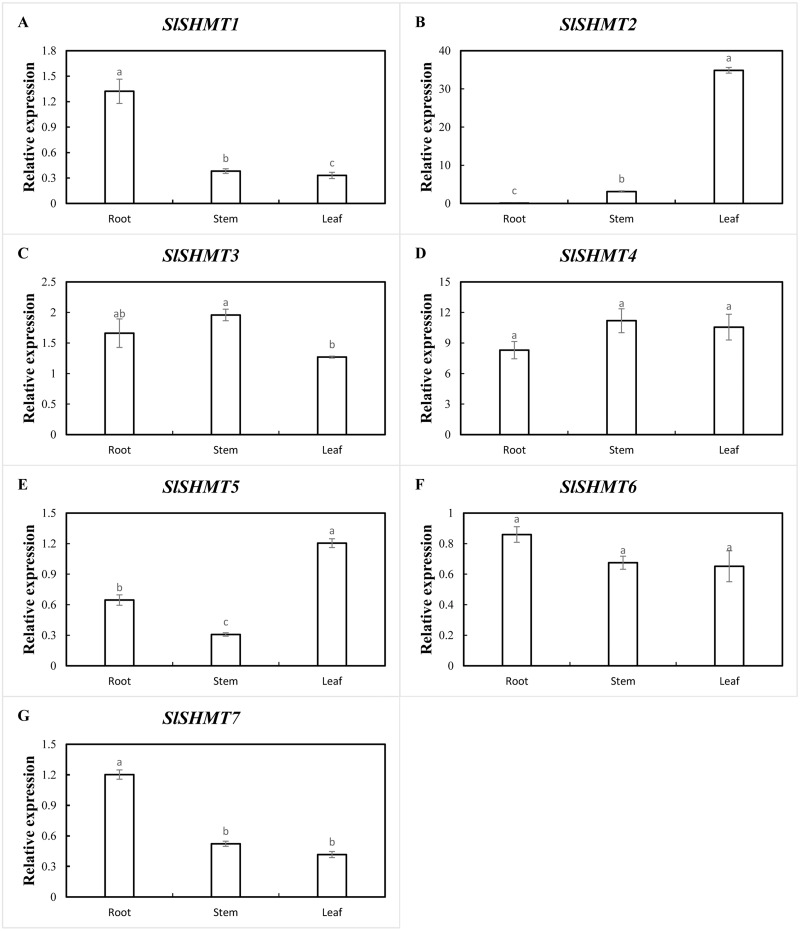
Expression levels of *SlSHMT* genes of different plant organs in vegetative growth period. The expression patterns of *SlSHMT1*-*SlSHMT7* in different tissues are shown in A–G, respectively. Error bars represent the standard error (SE) of three replicates. The relative expression of each gene in different tissues is expressed as mean ± SE (*n* = 3). Bars with different lowercase letters were significantly different by Duncan’s multiple range tests (*p* < 0.05).

**Figure 9:**
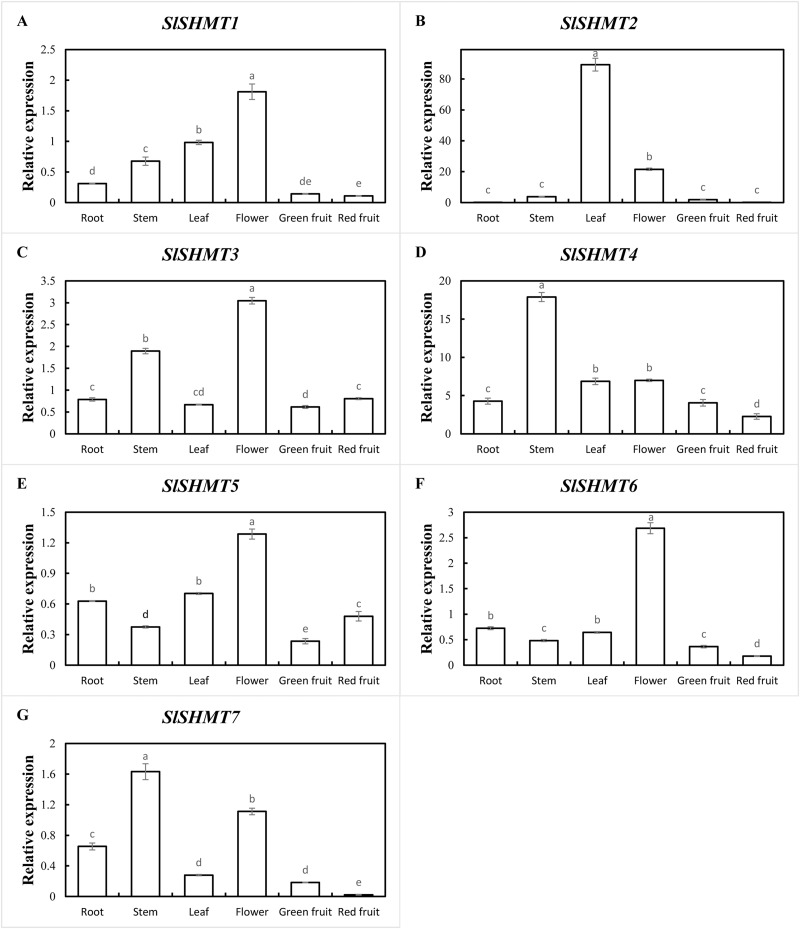
Expression levels of *SlSHMT* genes of different organs in reproductive growth period. The expression patterns of *SlSHMT1*-*SlSHMT7* in different tissues are shown in A–G, respectively. Error bars represent the standard error (SE) of three replicates. The relative expression of each gene in different tissues is expressed as mean ± SE (*n* = 3). Bars with different lowercase letters were significantly different by Duncan’s multiple range tests (*p* < 0.05).

In order to determine the expression specificity of tomato *SHMT* genes in different growth stages and organs, qRT-PCR was used to detect the expression levels of *SlSHMT* genes in different organs of tomato during vegetative and reproductive growth stages. In vegetative growth stage, *SlSHMT1* and *SlSHMT7* are highly expressed in root. The expressions of *SlSHMT2* and *SlSHMT5* are most abundant in leaf. However, the expression levels of *SlSHMT4* and *SlSHMT6* in root, stem and leaf are similar. The expression level of *SlSHMT3* gene in stem is relatively high (Fig. 8). In reproductive growth stage, *SlSHMT1*, *SlSHMT3*, *SlSHMT5* and *SlSHMT6* are highly expressed in flower. *SlSHMT4* and *SlSHMT7* are mainly expressed in stem. *SlSHMT2* gene is highly expressed in leaf (Fig. 9). The results show that *SlSHMT* genes might play special roles in different growth stages and different plant organs.

### Expression analysis of tomato *SHMT* genes under different treatments

**Figure 10:**
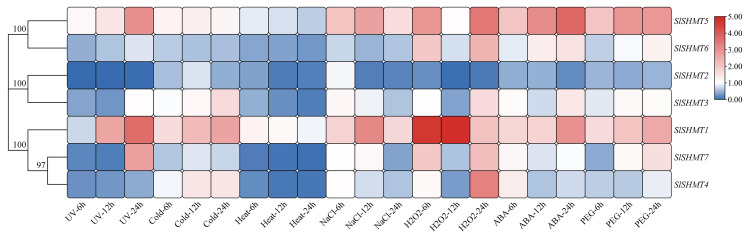
Expression levels of *SlSHMT* genes under UV radiation, cold, heat, NaCl, ABA, H_2_O_2_ and drought (PEG) treatments. Seedlings were treated with 253.7 nm UV radiation, 4 °C Cold, 40 °C Heat, 200 mM NaCl, 100 mM ABA, 10% (w/v) H_2_O_2_ and 20% (w/v) PEG. The color scale represents the folding changes normalized by log2 transformed data. Blue represents downregulated genes and red represents upregulated genes.

The correlation of plant SHMTs with biotic and abiotic stress defenses has long been discovered. For further analyze the response of tomato SHMT members to abiotic stresses and plant to hormones, qRT-PCR experiments were carried out and TBtools software was used to draw clustering heat map of *SlSHMT* gene expression levels under different treatments (Fig. 10 and File S5). The results show that expression levels of *SlSHMT* genes are different under different treatments. In terms of *SlSHMT1*, there is almost no change of expression level under heat treatment, but it is significantly up-regulated under UV radiation, cold, ABA and PEG treatments, reaching the highest expression level at 24 h. *SlSHMT1* is also significantly up-regulated under NaCl and H_2_O_2_ treatments, obtaining the highest expression level at 12 h. For *SlSHMT2*, its transcription level exhibits a downward trend under all treatments. *SlSHMT3* is slightly down-regulated under UV radiation, heat and NaCl treatments, and slightly up-regulated under cold, H_2_O_2_ and ABA treatments, but almost unchanged under PEG treatment. *SlSHMT4* is mildly down-regulated by UV radiation, heat, NaCl and PEG treatments, and slightly up-regulated under cold treatment, while the expression level of *SlSHMT4* under H_2_O_2_ and ABA are complicated. *SlSHMT5* is up-regulated under UV radiation, cold, NaCl, H_2_O_2_, PEG and ABA treatments but down-regulated under heat treatment. *SlSHMT6* is mildly down-regulated by UV radiation, cold, heat, NaCl and PEG treatments, but slightly up-regulated under ABA and H_2_O_2_ treatments. *SlSHMT7* is down-regulated and then up-regulated under UV radiation and PEG treatments, reaching the highest expression level at 24 h. Meanwhile, *SlSHMT7* is slightly down-regulated by heat treatment, and its expression does not change under other treatments.

## Discussion

SHMT catalyzes the conversion of serine to glycine and take part in cellular metabolism through providing one-carbon units for many biosynthetic reactions in many organisms (Bauwe & Kolukisaoglu, 2003). SHMT also acts as an indispensable role in the photorespiratory pathway of aerobic photosynthetic organisms (Waditee-Sirisattha et al., 2017). In addition, SHMT is widely distributed in plants and plays an active role in regulating plant stress resistance (Fang et al., 2020). Genes encoding *SHMT* have been identified in many higher plants. It has been shown that 18, 7 and nine *SHMT* genes are present in soybean (Lakhssassi et al., 2019), *A. thaliana* (Zhang et al., 2010) and *P. trichocarpa* (Li & Cheng, 2020), respectively. However, the *SHMT* gene family in tomato has not been studied in detail. In the study, we identified 7 *SHMT* genes in the tomato genome and they are randomly distributed on six chromosomes of tomato (Table 1, Fig. 5). Thus, the difference in genome size may lead to diverse amounts of SHMT family members (Xie et al., 2018). Soybean is the only tetraploid among the above plants, and its genome size is the largest. Soybean contains the most abundant SHMT members in comparison with the diploid tomato, *A. thaliana* and *Populus trichocarpa*, indicating a difference replication pattern of each kind of plants during the evolution.

We analyzed the gene structure of *SlSHMT* genes and found that the intron numbers of different *SlSHMT* genes are quite distant, ranging from three to 16 (Fig. 1). In parallel, the average exon length of *SlSHMT2*, *SlSHMT3*, *SlSHMT5* and *SlSHMT6* with multiple introns is relatively shorter than that of *SlSHMT1*, *SlSHMT4* and *SlSHMT7* with fewer introns, and the length of coding sequence in each *SlSHMT* gene does not show too much difference (Table 1). This phenomenon is similar with the *SHMT* genes in *A. thaliana* and soybean (Lakhssassi et al., 2019; Li & Cheng, 2020), demonstrating that SHMT genes may evolve from parts of members through alternative splicing. Here, the conserved regions of tomato SHMT proteins was also analyzed (Fig. 4). There are 10 conserved motifs found in SlSHMT proteins, and all SlSHMT proteins contain the typical SHMT (Pfam: PF00464) domain. Nevertheless, only four family members contain the tenth motif, suggesting different protein properties and functions between these SHMT proteins.

In order to further explore the relationship between tomato SHMT members and other species, a phylogenetic tree was constructed including SHMT members of tomato, *A. thaliana*, soybean and poplar. All the SHMTs members are divided into two groups and four subgroups namely ClassI-1, ClassI-2, ClassII-1 and ClassII-2, which is similar with previous studies in *A. thaliana*, soybean and poplar (Lakhssassi et al., 2019; Li & Cheng, 2020). Each subgroup contained at least one member of tomato SHMT (Fig. 2). In addition, the 7 predicted tomato *SHMT* genes are collinear with *SHMTs* of *A. thaliana*, soybean and poplar, and there are more than 4 homologous pairs between every two of these species (Fig. 3), indicating a conserved relationship within SHMTs in *A. thaliana*, soybean and poplar during evolution (Rajinikanth, Harding & Tsai, 2007). Further analysis of molecular weight, subcellular localization, motif composition and distribution of SlSHMTs shows that the SlSHMT members within each subgroup of ClassI-1, ClassI-2 and ClassII-1 are quite similar (Tables 1, 3 and Fig. 4). For example, the length of SlSHMT proteins in the same subgroup differs by no more than three amino acids (Table 1), suggesting that gene duplication events may occur at the species or lineage levels, and the proteins in each subgroup may exhibit a redundant function in plant growth and development (Wu et al., 2016).

In the current study, a part of the tomato SHMTs distribute in mitochondria, chloroplasts, and cytosol (Table 3), similar with that in *A. thaliana* and soybean (Bauwe & Kolukisaoglu, 2003; Lakhssassi et al., 2019). In plants, the serine produced by SHMT and GDC in mitochondria enters the cytosol and then serves as the primary one carbon donor to generate nucleotide, vitamin and amino acid, which is directed by the cytosolic SHMT (Rajinikanth, Harding & Tsai, 2007). The chloroplastic SHMT also presents a vital role for photoreception and for the biosynthesis concerning one carbon metabolism (Zhang et al., 2010). Thus, tomato SHMTs may also provide outstanding contributions on plant cellular metabolisms, which could be partially certified by the extensive expressions in root, stem, leaf and flower in tomato seedlings (Figs. 8, 9). Moreover, many mitochondrial SHMTs, such as AtSHMT1, OsSHMT1 and GmSHMT08, have been shown to participate in defense activities against biotic and abiotic stresses (Moreno, Martín & Castresana, 2005; Wang et al., 2015; Lakhssassi et al., 2020). We speculate that the mitochondrion-localized SlSHMT2 and SlSHMT3 may have similar functions with them, which was confirmed by a recent study in tomato (Ahammed et al., 2018). Unpredictably, the class II-1 members SlSHMT4 and SlSHMT7 are shown to localize in cytoskeletons (Table 3), which has not been reported in other plants so far, and their accurate function remains to be discovered. Meanwhile, the tomato SHMT members rarely distribute in nucleus.

In plants, *SHMT* genes have been identified in many tissues (McClung et al., 2000; Zhang et al., 2010; Lakhssassi et al., 2019), but the expression levels vary according to different *SHMT* members in different growth and development stages. In *A. thaliana*, *AtSHMT1* mainly distributes in leaf, stem and flower, and *AtSHMT1* mutation induces aberrant regulation of cell death and enhances susceptibility to pathogens and abiotic stress (Moreno, Martín & Castresana, 2005). *AtSHMT3* is mainly expressed in germinated seed, which potentially regulates metabolic fluxes in plastids (Zhang et al., 2010), while *AtSHMT4* transcripts accumulation is restricted to root in young seedlings (McClung et al., 2000). *BvSHMTa* is expressed in the leaf and root in sugar beet, and its expression level could be strengthened by salt stress (Kito et al., 2017). In soybean, most of the *GmSHMT* genes present a ubiquitous expression in all the tissues with some exceptions, while some of these members display irredundant responses during SCN infection (Lakhssassi et al., 2019). In the present study, all the tomato *SHMT* genes could be expressed in root, stem, leaf and flower, except for a very low expression of *SlSHMT2* in root, indicating that the enzyme activities of tomato SHMTs are essential for the growth and development of various growth stages. In addition, *SlSHMT2* transcripts is the most abundant ones in all the analyzed tissues, and *SlSHMT2* is specifically expressed in leaf, which is similar with the tissue localization of *AtSHMT1* and *OsSHMT1* (Moreno, Martín & Castresana, 2005; Wang et al., 2015). Thus, the molecular function of SlSHMT2 in green leaves may be conserved in tomato, rice and *A. thaliana*. Further, *SlSHMT1*, *SlSHMT3*, *SlSHMT5* and *SlSHMT6* exhibit the most abundant expression levels in flower, while expressions of *SlSHMT4* and *SlSHMT7* are relatively higher in stem in reproductive growth stage. Thus, SHMT members may function redundantly in leave, stem and flower to support the metabolism activities concerning fruit development, and the specific mechanism needs to be explored in detail. Simultaneously, we detected a mildly and redundantly expression of tomato *SHMT* genes in the fruit tissues, which was divergent with the reported expressions of SHMT members in seeds (Zhang et al., 2010; Lakhssassi et al., 2019), suggesting a different regulation mechanism of SHMTs in fruits and the mature seeds.

The *cis*-acting elements in the promoter region of genes have long been studied as the binding sites of specific transcription factors, which help to modulate the initiation of genes transcription (Carrier et al., 2020). Up to now, many of the *cis*-acting elements have been well characterized and classified into different groups (Hadiarto & Tran, 2011). We have identified a number of *cis*-acting elements in the promoter regions of tomato *SHMT* genes, including abiotic stresses-related LTR, ARE, MBS and TC-rich repeats; hormones-related CGTCA-motif, P-box, TCA-element, TGACG-motif, TGA-element and ABRE; and light-related Circadian and G-box (Table 5). The widely present of these *cis*-acting elements suggests a crucial role of *SlSHMT* members in response to abiotic stresses and hormone stimulations. For example, expression of *SlSHMT5* is up-regulated under PEG and ABA treatments, which was consistent with the distributions of drought and ABA response elements (MBS, ABRE) in *SlSHMT5* (Fig. 10). However, some conflicting results also appeared in our further study. Both *SlSHMT4* and *SlSHMT7* contain AREB binding site in their promoter regions (Fig. 7), but neither of them responds to ABA treatment. *SlSHMT3* has been found to mediate plant basal defence against *Pseudomonas syringae* in a salicylic acid (SA)-dependent manner (Ahammed et al., 2018), but it does not have a SA response element (TCA-element). This phenomenon was also found in other gene families previously (Gao et al., 2021). In addition, the restricted analyzed tissue, growth stage and treated period may influence the judgment of their relationships. Thus, more explorations are yet to be done to uncover the detailed relationships between different responses of *SlSHMT*s and the corresponding *cis*-acting elements.

As an important house-keeping regulator of plant growth and development, the response of SHMT gene family members to adverse environmental conditions has been reported in many species. In our study, *SlSHMT* genes could respond to variety kinds of circumstance stimuli. In detail, *SlSHMT1* gene expression is increased by UV, cold, salt, H_2_O_2_ and PEG stress. Expression level of *SlSHMT5* is significantly enhanced by UV, salt, H_2_O_2_ and PEG treatment. The alleviation of salt stress by SHMT members have been discovered in many higher plants as well as in the procaryotic organism cyanobacterium. In *A. thaliana*, a ubiquitin-specific protease UBP16 interacts with AtSHMT1 to stabilize it, and they cooperate to increase Na^+^/H^+^ antiport activity under salt stress (Zhou et al., 2012). In rice, the overexpression level of *OsSHMT3* was found to be up-regulated in a salt-tolerant line ‘CSR27′(Mishra et al., 2016; Mishra et al., 2019). Overexpression of *ApSHMT* (a gene isolated from cyanobacterium) in *Escherichia coli.* could increase the accumulation of glycine betaine, which leads to an intensive tolerance of the transgenic cells (Srivastava et al., 2011; Waditee-Sirisattha et al., 2012). Plant SHMTs were also found to relieve damages of chilling and drought through maintaining redox homeostasis and regulating stomatal closure (Liu et al., 2019a; Liu et al., 2019b; Fang et al., 2020). Interestingly, heat stress reduces the expression levels of all *SlSHMT*s except for *SlSHMT1*. Moreover, *SlSHMT2* is down-regulated by all the treatments analyzed. The similar phenomenon was also reported in pitaya (Hylocereus undatus), *A. thaliana* and rice (Xu & Huang, 2010; Barkla et al., 2014; Fan et al., 2014; Chen et al., 2020a; Chen et al., 2020b). Plant SHMTs influence gene replication through regulating nucleotide synthesis, and the decrease of SHMT activities under abiotic stresses may reduce the probability of inaccurate replication events (Xu & Huang, 2010). The responses of other *SlSHMT* members varied in different conditions, indicating a redundant function of tomato SHMTs in resisting environmental stresses. Also, the diverse responses to abiotic stresses may due to the evolutionary discordance within *SHMT* gene members in different species.

## Conclusions

In general, seven *SHMT* genes in tomato genome were identified in the present study. Physicochemical properties, gene and protein structures, and evolutionary relationships may predict structure and function similarity as well as divergence among tomato SHMTs and their orthologs in other dicotyledonous members. What’s more, subcellular location and tissue-specific analysis shows that *SlSHMT* members conduct a pivotal house-keeping function through regulating cellular metabolisms during plant growth and development. Last but not the least, we provide evidence that tomato SHMTs participate in alleviating various abiotic stresses and hormone responses. In summary, this study attempts to support further studies on the involvement of the *SlSHMT* gene family in growth regulation and stress response in tomato, and provide theoretical foundation for further exploration of the function of plant SHMT members.

##  Supplemental Information

10.7717/peerj.12943/supp-1 Supplemental Information 1 QRT-PCR primers for expression analysis of *SHMT* gene family in *Solanum lycopersicum*

10.7717/peerj.12943/supp-2 Supplemental Information 2 The conserved SHMT domains found in seven SlSHMTs which confirmed that all the seven genes analysis belong to SHMT gene family

10.7717/peerj.12943/supp-3 Supplemental Information 3 *SHMT* genes used to construct the phylogenetic tree

10.7717/peerj.12943/supp-4 Supplemental Information 4 *Cis*-acting elements of *SlSHMTs* in promoter region

10.7717/peerj.12943/supp-5 Supplemental Information 5 Raw data of qRT-PCR experiment in analyzing responses of tomato SlSHMTs to different abiotic stresses and hormone stimulus

## Additional Information and Declarations

### Data Availability

The following information was supplied regarding data availability: The raw measurements are available in the Supplementary Files.
