# Supplementary material for: Genome-wide identification and expression analysis of serine hydroxymethyltransferase (SHMT) gene family in tomato (Solanum lycopersicum)
Source: PeerJ. 2022 Feb 10;10:e12943. doi: 10.7717/peerj.12943 (PMC8841039; doi:10.7717/peerj.12943)
Supplement: Supplemental Information 1 [file peerj-10-12943-s001.docx]

Supplementary file 1. qRT-PCR primers for expression analysis of *SHMT* gene family in *Solanum lycopersicum*.

| Gene name | Primer sequence (5^，^to 3^，^) | |
| --- | --- | --- |
| *SlSHMT1* | F:GAGAGAGTGCGGATGAGGAGAG | R: CTAACCGCATTTCTTCGTGACTC |
| *SlSHMT2* | F: CCTGAGATTGCTGACATTATTGAG | R:CGTTTCTGGCATAAGGTTTCTG |
| *SlSHMT3* | F: CAATACAAGTCGTCTTTGCCTAATC | R:CAAGACCCTTCCATTGCCTAG |
| *SlSHMT4* | F:GCTTTAACCAACAAATACTCAGAGG | R: GAATAAGGCTGAACATTAACACCC |
| *SlSHMT5*  *SlSHMT6*  *SlSHMT7*  *SlActin* | F: GAGTCGGGTAAAGTTATGCTCTGTC  F:GCCTCCTTCTTCTGTTTCTGTTC  F: CGCTGTAATTGAAGCTCTAGGC  F: AATGAACTTCGTGTGGCTCCAGAG | R: CTCCTAAACTGACGCTGCTTCTC  R: CCGCCTCCATCACTGCTC  R: CCTGAATATGGCTGAACATTAACAC  R: ATGGCAGGGGTGTTGAAGGTTTC |
