# Supplementary material for: Genome-wide identification and expression analysis of serine hydroxymethyltransferase (SHMT) gene family in tomato (Solanum lycopersicum)
Source: PeerJ. 2022 Feb 10;10:e12943. doi: 10.7717/peerj.12943 (PMC8841039; doi:10.7717/peerj.12943)
Supplement: Supplemental Information 2 [file peerj-10-12943-s002.docx]

Supplementary file 2. Domains in seven SlSHMTs.

| Gene | ID | Accession | Clan | Start-End | E-value | |
| --- | --- | --- | --- | --- | --- | --- |
|  |  |  |  |  | Ind. | Cond. |
| *SlSHMT1* | *Solyc01g104000.3.1.ITAG3.2* | PF00464.19 | CL0061 | 137-542 | 6.6e-165 | 7.4e-169 |
| *SlSHMT2* | *Solyc02g091560.3.1.ITAG3.2* | PF00464.19 | CL0061 | 56-454 | 1.6e-211 | 9.0e-216 |
| *SlSHMT3* | *Solyc04g076790.3.1.ITAG3.2* | PF00464.19 | CL0061 | 55-453 | 4.1e-209 | 2.3e-213 |
| *SlSHMT4* | *Solyc05g053810.3.1.ITAG3.2* | PF00464.19 | CL0061 | 12-412 | 1.0e-187 | 5.6e-192 |
| *SlSHMT5* | *Solyc08g065490.3.1.ITAG3.2* | PF00464.19 | CL0061 | 78-468 | 3.3e-191 | 1.8e-195 |
| *SlSHMT6* | *Solyc12g095930.2.1.ITAG3.2* | PF00464.19 | CL0061 | 81-471 | 3.1e-189 | 3.4e-193 |
| *SlSHMT7* | *Solyc12g098490.2.1.ITAG3.2* | PF00464.19 | CL0061 | 12-412 | 6.1e-185 | 3.4e-189 |
