# Supplementary material for: Genome-wide identification and expression analysis of serine hydroxymethyltransferase (SHMT) gene family in tomato (Solanum lycopersicum)
Source: PeerJ. 2022 Feb 10;10:e12943. doi: 10.7717/peerj.12943 (PMC8841039; doi:10.7717/peerj.12943)
Supplement: Supplemental Information 3 [file peerj-10-12943-s003.docx]

Supplementary file 3. *SHMT* genes used to construct the phylogenetic tree.

| Species | Gene names | Gene ID | Database |
| --- | --- | --- | --- |
| *Arabidopsis thaliana* | *AtSHMT1* | *AT4G37930* | TAIR |
|  | *AtSHMT2* | *AT5G26780* | Phytozome |
|  | *AtSHMT3* | *AT4G32520* |  |
|  | *AtSHMT4* | *AT4G13930* |  |
|  | *AtSHMT5* | *AT4G13890* |  |
|  | *AtSHMT6* | *AT1G22020* |  |
|  | *AtSHMT7* | *AT1G36370* |  |
|  |  |  |  |
| *Glycine max* | *GmSHMT02m* | *Glyma.02g217100* | (*Lakhssassi et al., 2019*) |
|  | *GmSHMT04n* | *Glyma.04g254300* |  |
|  | *GmSHMT05c* | *Glyma.05g152100* |  |
|  | *GmSHMT06n* | *Glyma.06g107800* |  |
|  | *GmSHMT08c* | *Glyma.08g108900* |  |
|  | *GmSHMT08m* | *Glyma.08g274400* |  |
|  | *GmSHMT08n* | *Glyma.08g187800* |  |
|  | *GmSHMT09m* | *Glyma.09g202000* |  |
|  | *GmSHMT09n* | *Glyma.09G184300* |  |
|  | *GmSHMT12c* | *Glyma.12g159200* |  |
|  | *GmSHMT12n* | *Glyma.12g170300* |  |
|  | *GmSHMT13c* | *Glyma.13G077700* |  |
|  | *GmSHMT13ch* | *Glyma.13g222300* |  |
|  | *GmSHMT14m* | *Glyma.14g184500* |  |
|  | *GmSHMT15c* | *Glyma.15G090000* |  |
|  | *GmSHMT15ch* | *Glyma.15g089900* |  |
|  | *GmSHMT16c* | *Glyma.16G108100* |  |
|  | *GmSHMT18m* | *Glyma.18g150000* |  |
|  |  |  |  |
| *Populus trichocarpa* | *PtrSHMT1* | *Potri. 001G212000.3* | Phytozome |
|  | *PtrSHMT2* | *Potri. 001G320400.2* | (*Bing et al.,2020*) |
|  | *PtrSHMT3* | *Potri. 002G090200.1* |  |
|  | *PtrSHMT4* | *Potri. 002G109200.5* |  |
|  | *PtrSHMT5* | *Potri. 005G170800.1* |  |
|  | *PtrSHMT6* | *Potri. 006G232300.2* |  |
|  | *PtrSHMT7* | *Potri. 008G002900.14* |  |
|  | *PtrSHMT8* | *Potri. 010G254700.15* |  |
|  | *PtrSHMT9* | *Potri. 017G059300.1* |  |
|  |  |  |  |
| *Solanum lycopersicum* | *SlSHMT1* | *Solyc01g104000.3.1.ITAG3.2* | Phytozome |
|  | *SlSHMT2* | *Solyc02g091560.3.1.ITAG3.2* |  |
|  | *SlSHMT3* | *Solyc04g076790.3.1.ITAG3.2* |  |
|  | *SlSHMT4* | *Solyc05g053810.3.1.ITAG3.2* |  |
|  | *SlSHMT5* | *Solyc08g065490.3.1.ITAG3.2* |  |
|  | *SlSHMT6* | *Solyc12g095930.2.1.ITAG3.2* |  |
|  | *SlSHMT7* | *Solyc12g098490.2.1.ITAG3.2* |  |
